# MOLGENIS catalogue

**DOI:** 10.1186/2043-9113-5-S1-S8

**Published:** 2015-05-22

**Authors:** Morris Swertz, David van Enckevort, Chao Pang

**Affiliations:** 1 Department of Genetics, University Medical Center Groningen, University of Groningen, 9700RB Groningen, the Netherlands; 2 Department of Epidemiology, University of Groningen, University Medical Center Groningen, Groningen, the Netherlands

## Characterisation

Tool, biobanking, meta-data, data, miabis, open source.

## Description

MOLGENIS/catalogue is a generic toolbox for building biobank and study catalogues and is used in BBMRI-NL, EU-BioSHaRE, EU-BioMedBridges, LifeLines, CTMM/TraIT, Durrer Center, PALGA NL pathology network. The catalogue can host four levels of information:

1) Biobank/study descriptions using custom or MIABIS standard of BBMRI-ERIC format;

2) Data schema/data dictionary of data elements;

3) Aggregate data/sample availability counts and;

4) Individual level data ready for analysis.

**Figure 1:**
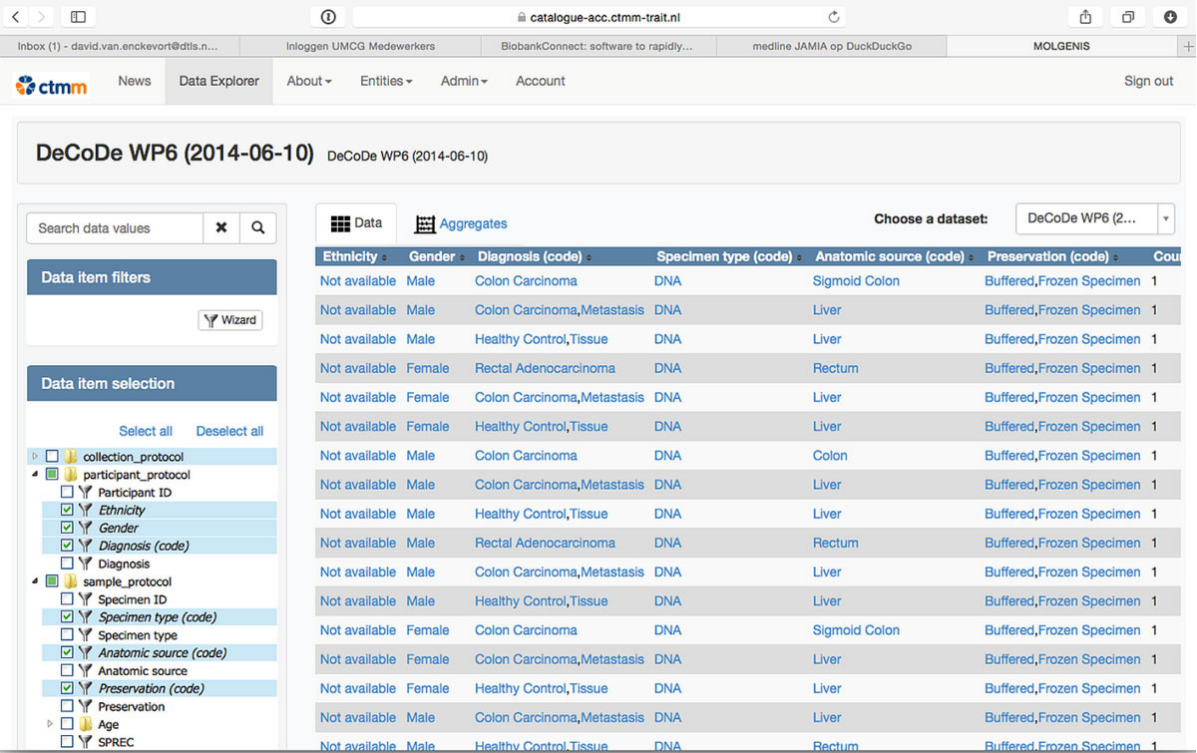
MOLGENIS/catalogue data explorer for the DeCoDe study. Users can filter, aggregate and drill down on available data.

Increasingly bigger datasets are required for epidemiological and genetic analysis; hence it has become important to enable pooling of data from multiple biobanks. Therefore, the catalogue also comes with BiobankConnect, a tool to rapidly match data elements across studies and biobanks based on lexical matching and ontologies [1]. MOLGENIS/catalogue is build on the open source MOLGENIS platform [2] and offers pre-build components that allow users to upload data in a simple Excel format and supports any data model through a meta-data definition in the Excel file; to visualize the data in aggregated or tabular form; to share securely data through a comprehensive security model and to integrate data from different domains (Figure 1).

## Status of development

Stable/production ready; version 1.2.0.

## Users

10 known installations.

## Links

http://github.com/molgenis/molgenis, http://www.molgenis.org, https://www.dropbox.com/s/bez1r4lq5q69o9e/Swertz%20ECRIN%20Dussseldorf%20catalogue%202014_05_26.pptx.pdf.pdf
